# Rock River Union Medical Society

**Published:** 1859-02

**Authors:** G. W. Phillips

**Affiliations:** Secretary


### PROCEEDINGS OF MEDICAL SOCIETIES.

### ROCK RIVER UNION MEDICAL SOCIETY.

The annual meeting of this society was held at Dixon, June 9th, 1858. The members assembled at Union Hall.

The President, Dr. A. S. Hudson, of Sterling, called the meeting to order.

The Secretary read the proceedings of the last meeting, which, on motion, were accepted.

The following gentlemen were admitted members of the Society:

Dr. Wynn, of Dixon, proposed by Dr. Abbott;

Dr. Dewitt C. Warner, of Dixon, proposed by Dr. Phillips;

Dr. D. Williams, of Sterling, proposed by Dr. A. S. Hudson.

Treasurer's report read and accepted.

On motion of Dr. N. W. Abbott, each member was assessed fifty cents.

Dr. Abbott, who was appointed at a previous meeting of the Society to make a report upon the "Uses of Veratrum Viride," stated that he had not prepared a written report, but would simply state his views of, and his experience with, the remedy. He believed veratrum viride to be the most potent remedy we possess, for reducing the pulse, in inflammatory and febrile diseases. He gave it in typhoid cases, even where there is a weak pulse, with great debility. He believed all diseases to be diseases of debility—all remedies are stimulants. Veratrum promotes all the secretions—controls the action of the heart by its direct specific effect.

On motion of Dr. G. W. Phillips, Dr. A. T. Hudson, of Lyons, Iowa, was invited to participate in the proceedings of the Society.

The retiring President, Dr. A. S. Hudson, of Sterling, as he was not prepared with a written address, would make the subject of his valedictory, the "Powers and Uses of Veratrum Viride." He had had but little experience in the use of the remedy; was at first disappointed in its effects, although he gave it in inflammatory cases, as to the strength and doses of the tincture, as directed by Dr. Norwood, but failed to control the action of the heart. The cause of not getting the effect, he thought, was the violence of the disease, the dose of the tincture being too small, and probably the variable strength of the article used. Dr. H. remarked that veratrum viride grown at the South is much better and stronger than that grown at the North. The rule which should guide us in administering the remedy, should be its effects, the same as should guide us in giving all remedies; if the effect cannot be produced by a certain dose, given every three hours, give the same every hour or two, or increase the dose. Dr. H. believed the remedy to stand on a par with the lancet; that it controls inflammation by reducing arterial action, if promptly and vigorously given; but ineffectual in typhoid cases, and positively injurious in such cases, attended with great prostration, and a weak, quick pulse. In this connection Dr. H. spoke of puerperal peritonitis; that in inflammation of the peritonæum, the membrane secretes gas, causing tympanitis, and that this symptom is pathognomonic of disease of that membrane; in inflammation of the proper substance of the uterus, there is no tympanitis, but always if the peritoneal covering of the organ is inflamed. Dr. H. made mention of the treatment of puerperal peritonitis by certain physicians of the city of New York, which is the use of opium and veratrum viride, the opium to relieve the pain and frequent breathing, given in large doses so as to produce narcosis, and the veratrum to control arterial action. He believed that opium not only relieved pain, but exerted a curative effect upon the inflammation.

The subject of Medical Intercourse, which had been discussed at the previous meeting, was introduced by Dr. A. S. Hudson, who contended that our patients should not be informed of the names of the remedies prescribed by the physician; that frequently, through the prejudices of patients against a certain remedy, the physician's prescription is not carried out; that the practice of thus informing our patients, is injurious to them as well as ourselves; that it brings disrespect and dishonor to the profession.

Dr. J. B. Nash made some remarks, sustaining these views. No resolution was offered or adopted upon the subject. In this connection, Dr. A. T. Hudson, of Lyons, made some remarks in regard to disguising the taste of medicines, as the disagreeable taste was frequently the cause of prejudice against remedies. Dr. H. stated that, in Lyons, the physicians were in the habit of giving their medicine in wafers.

To cover the taste of quinine, Dr. J. K. Eberle gives it in liquorice.

Dr. Phillips stated that he was in the habit of giving quinine in strong, hot tea or coffee, or, by adding a little tannin to the quinine, and dissolving it in water; either of which methods covers the taste most effectually.

Remarks were introduced, by Dr. Nash, in regard to the alterative effects of tart. antimony; he had been in the habit of curing enlarged spleen, caused by a long continuance of an intermittent, by giving the remedy in small doses, continued for a long time.

Dr. Abbott remarked that he never used tart. antimony, except as an emetic; he believed it to be a dangerous remedy for children; he never used it in the pneumonia of young children; and that the only effect he knew of its possessing, except its emetic, was its sedative action upon the circulation.

Dr. Phillips remarked, that tart. antimony had not only an emetic and sedative effect, but a contra-stimulant effect upon the mucous membrane of the stomach and bowels, and that this is its most valuable property, and the most to be relied upon in the treatment of pneumonia. Dr. P. remarked, that he had used the remedy in the pneumonia of young children, even nursing children, without producing bad effects upon the mucous membrane; the reason that physicians found it to produce inflammation of the mucous membrane of the alimentary canal, and probably destroyed their little patients, was that they did not discriminate their cases. Tart. ant. cannot be used in all cases of pneumonia of children; if there be a co-existing inflammation of the mucous membrane, with the pneumonia, the remedy should not be used; but if no complication of this nature exists, it is safe to use tart. antimony. It would not be safe to use tart. ant. in pneumonia, complicated with miasmatic fever, unless the inflammatory element predominated, and the accompanying fever is, in a great degree, symptomatic. Cases of pneumonia are met with, with the physical symptoms of that disease, but with an accompanying fever, that is strictly miasmatic; the use of tart. ant. in a case like this would fail to meet the disease; a very different treatment is required; quinine, in free doses, must be used, to break down the fever, when the local disease will readily yield to the use of mild remedies.

Dr. Geo. W. Hewitt exhibited, before the Society, a patient with tænia, as he supposed; the patient, a man some thirty-five or forty years of age, first felt symptoms of the disease last autumn. The symptoms, at this time, are as follows: The patient has tolerable health; he thinks he is not quite so fleshy as in health; appetite variable; bowels generally regular; the patient describes his sensations, as of something moving about in the abdomen, sometimes with a rough, dragging feeling, or a sensation of something biting. Dr. H. has given the patient freely of ol. terebinth, to destroy the worm; the patient says the cathartic drives the worm lower down, into a bunch; he thinks he has felt it in the rectum, and he says he can indicate the part of the abdomen in which it lies; he says it is now low down in the right iliac region; upon percussion over this part, a marked dullness is found over the entire region, nearly. The patient, at one time, dosed himself with aloes and whisky; this, he said, caused the worm to pass low down, and "made him very vicious," causing it to move about a great deal; when he has been fasting, he thinks the worm rises to the stomach, causing nausea. Many of the patient's sensations may be magnified. The worm appears to be wadded up, either in the cecum or lower part of the ileum.

Some members expressed their doubt as to its being a tape worm. Dr. A. S. Hudson mentioned the case of a boy, in Michigan, who vomited up a frog; he remembered to have drank at a spring, and the inference was that he swallowed a polliwog.

The treatment advised was, to give cathartic remedies, to pass the worm into the large intestine or rectum, and then to endeavor to hook it out. Another course advised was, to let the patient fast, until the worm rises to the stomach, then to give an emetic of sulphate of zinc.

The Society next proceeded to elect officers.

Dr. J. A. Jackson, of Amboy, was elected *President*,
Dr. Geo. W. Hewitt, of Franklin Grove, *Vice-President*,
Dr. Moses M. Roger, of Sterling, . . *Treasurer*,
Dr. G. W. Phillips, unanimously re-elected *Secretary*.

A suggestion was made by Dr. Phillips, and on motion of Dr. Abbott, adopted, that a Committee be appointed upon Practical Medicine; a schedule of questions made out, and sent to each member of the Society, with a request that each shall make a report to the Committee upon each subject.

On motion, Dr. G. W. Phillips, as Chairman, and Dr. A. S. Hudson, were appointed said Committee. On motion of Dr.

D. Williams, the Committee were authorized to select the schedule.

On motion of Dr. Phillips, Dr. A. S. Hudson was appointed to make a report, at the next semi-annual meeting, upon "The Curative Effects of Opium, in Inflammatory and Febrile Diseases."

On motion of Dr. Nash, Dr. Oliver Everett was appointed to deliver the popular address at the next semi-annual meeting.

On motion, Dr. A. S. Hudson was appointed as delegate to the "American Medical Association."

On motion, Drs. Abbott and Jackson were appointed delegates to the State Medical Society.

On motion of Dr. Abbott, Amboy was selected as the place for the next semi-annual meeting, and Sterling for the annual meeting.

A suggestion was made by Dr. A. T. Hudson, which, on motion of Dr. Abbott, was adopted; that the Secretary confer with other Medical Societies, for the purpose of gaining their co-operation to memorialize the next legislature to so amend the exemption laws, that no class of property shall be exempt against physician's fees.

On motion of Dr. Abbott, the Secretary was authorized to send a copy of the proceedings of this meeting, for publication in the *Chicago Medical Journal.*

On motion, adjourned to 7½ o'clock P.M.

*Evening Session.*—Dr. Jackson in the chair. Dr. A. S. Hudson delivered the annual Address: subject, "The Correlation of Life and Death."

Adjourned to meet at the semi-annual session, the time of such meeting to be fixed by the President and Secretary; the members to be informed of the time and place by circular.

G. W. PHILLIPS, M.D., *Secretary.*

